# Perioperative anesthetic management for cesarean delivery of severe Wilson’s disease with liver failure: a case report

**DOI:** 10.1186/s40981-019-0294-2

**Published:** 2019-11-13

**Authors:** Kana Saito, Eiko Onishi, Jun Itagaki, Noriko Toda, Azusa Haitani, Masanori Yamauchi

**Affiliations:** 1 Department of Anesthesiology, Tohoku University Hospital, 1-1 Seiryo-machi, Aoba-ku, Sendai, Miyagi 980-8574 Japan; 2 Department of Anesthesiology, Tohoku Rosai Hospital, 4-3-21 Dainohara, Aoba-ku, Sendai, Miyagi 981-8563 Japan

**Keywords:** Wilson’s disease, Coagulopathy, Cesarean delivery, Rotational thromboelastometry

## Abstract

**Background:**

Wilson’s disease is a rare autosomal recessive disorder affecting copper metabolism, which presents liver and brain dysfunction caused by abnormal copper accumulation. We report a patient who showed exacerbation of liver failure during pregnancy.

**Case presentation:**

A 24-year-old woman with Wilson’s disease was scheduled for emergency cesarean delivery at 30 weeks of gestation. The patient exhibited severe coagulopathy and prominent body weight gain (+ 30 kg) caused by systemic edema and ascites. We decided to perform emergency cesarean delivery under general anesthesia. We used platelet concentrates, cryoprecipitate, and fibrinogen concentrate. Intraoperative hemorrhage was well controlled. On the 15th postpartum day, weight was reduced by 20 kg and liver function had improved. She and her baby were discharged without complications.

**Conclusions:**

The appropriate continued treatment of Wilson’s disease and supplementation of coagulation factors and/or platelets when indicated greatly increase the likelihood of a successful pregnancy, even in patients with liver failure exacerbation.

## Background

Wilson’s disease (WD) is a rare autosomal recessive disorder whose main symptom is abnormal copper accumulation mainly in the liver and brain [1, 2]. WD shows a wide range of clinical manifestations, including hepatic disease, neurological abnormality, psychiatric disorder, and Kayser-Fleischer rings (copper deposition on the periphery of the cornea) [3]. Liver damage induces chronic hepatitis, liver cirrhosis, and fulminant hepatic failure, causing severe coagulopathy, encephalopathy, and hemolytic anemia.

Several reports on pregnant patients with WD have been reported, wherein patients with well-controlled WD have had successful pregnancy outcomes with continued disease treatment and close monitoring [1, 2, 4–7]. In recent retrospective study of pregnancy in WD, liver dysfunction during pregnancy has been observed in 6% of patients, and it had normalized after delivery [7]. However, the anesthetic management of severe WD with pregnancy has not been reported.

We report a patient showing liver failure exacerbation during pregnancy. She was at high risk of peripartum hemorrhage due to thrombocytopenia, hypofibrinogenemia, and obstetric disseminated intravascular coagulation (DIC). She also showed a gain of 30 kg body weight and dyspnea due to severe systemic edema and ascites. Preoperative platelet transfusion and supplemental coagulation factor including cryoprecipitate and fibrinogen concentrate were effective to prevent peripartum hemorrhage and to avoid a large volume of transfusion.

## Case presentation

A 24-year-old woman (height, 169.2 cm; body weight, 79.2 kg) with WD was scheduled for emergency cesarean delivery at 30 weeks of gestation.

The patient was diagnosed with WD at 3 years of age. Her liver function had deteriorated severely enough to require liver transplantation at 20 years of age. However, anti-copper therapy improved her liver function and was continued during pregnancy. She was hospitalized at 25 weeks of gestation for thrombocytopenia and fetal growth restriction (FGR) caused by placental insufficiency. Aspartate aminotransferase (AST) and alanine aminotransferase (ALT) serum levels were normalized during pregnancy, while antithrombin III (AT-III) levels were significantly lower (Table 1). On hematological evaluation, performing anticoagulation therapy with AT-III concentrate was suggested to prevent thrombophilia-induced placental dysfunction.

**Table 1:** Peripartum data of liver function, coagulation, copper metabolism and body weight

		Normal	Gestation weeks			Before CS	After CS			
		Ranges	25 weeks	29 weeks	30 weeks	30 weeks and 3 days		POD1	POD4	POD 40
Hemoglobin	g/dL	11.6-14.8	13.2	12.7	11.7	10.1	8.2	9.8	10.5	11.3
Platelets	×10^3^/μL	158-348	78	70	57	101	72	75	65	143
Total bilirubin	mg/dL	0.4-1.5	0.8	0.7	1.0	0.8	0.6	0.8	1.4	1.2
Albumin	g/dL	4.1-5.1	2.8	2.0	2.5	3.0	2.0	2.7	2.6	4.2
AST	IU/L	13-30	37	31	25	21	16	25	26	40
ALT	IU/L	7-23	27	19	14	14	9	11	12	29
ChE	U/L	201-421	52	43	33			89	86	109
PT	%	80-127	79.6	66.8	52.7	49.6	43.6	46.5	46.7	59.9
PT-INR		<1.15	1.11	1.2	1.31	1.38	1.46	1.42	1.40	1.26
APTT	sec	26.9-38.1	37.4	44.8	50.2	38.4	52.8	48.3	56.3	36.2
Fibrinogen	g/L	2.00-4.00	2.01	1.55	1.24	1.30	1.30	1.12	1.27	1.37
Antithrombin III	%	80-130	36	72	41	56	38	60	43	47
Serum copper	μg/dL	66-130	34						28	45
Ceruloplasmin	mg/dL	21-37	6.2						6.6	8.3
Body Weight	kg	49^a^	59.3	70	76.1	79.1	77.2	77.8	69.1	60.8

*CS* cesarean section, *POD* postoperative day, *AST* aspartate aminotransferase, *ALT* alanine aminotransferas, *ChE* cholinesterase, *PT* prothrombin time, *INR* international normalized ratio, *APTT* activated partial thromboplastin time

^a^before pregnancy

The patient’s body weight had rapidly increased by 1 kg/day with marked ascites and severe edema. She experienced dyspnea and could not ambulate due to dramatic weight gain. She was also diagnosed with obstetric DIC presenting with oliguria, thrombocytopenia, respiratory distress, liver dysfunction, and severe coagulopathy [8]. At 30 weeks of gestation, emergency cesarean delivery was performed under general anesthesia to improve her liver function. We performed supplementation with 10 units of platelet transfusion, 3 packs of cryoprecipitate, and 3 g fibrinogen concentrate.

In the operating room, a 16G venous catheter was inserted. We used rotational thromboelastometry (ROTEM® sigma, Tem Innovations GmbH, Munich, Germany), which showed normal coagulation function (Table 2). Under standard monitoring and bispectral index, we performed rapid sequence induction using 130 mg propofol, 0.05 mg remifentanil, and 70 mg rocuronium. Because her face and tongue were edematous and the Mallampati classification was III, intubation difficulties were expected. Therefore, we used a video laryngoscope (McGRATH MAC#3) for tracheal intubation. We maintained with oxygen in nitrous oxide (2:3) and 0.7% sevoflurane until delivery.

**Table 2:** ROTEM results before cesarean delivery

	FIBTEM (normal ranges)	EXTEM (normal ranges)	INTEM (normal ranges)	APTEM (normal ranges)
CT, seconds	59 (46–84)	54 (50–80)	142 (161–204)	53 (41–80)
CFT, seconds		108 (46–149)	108 (62–130)	112 (62–184)
Alpha angle, °	70	72 (63–83)	73 (66–77)	75 (60–80)
A5, mm	10 (5–20)	36 (32–52)	35 (33–52)	35 (28–50)
A10, mm	10 (6–21)	46 (43–63)	45 (43–62)	45 (39–61)
A20, mm	10 (6–21)	53 (52–70)	52 (50–68)	51 (48–68)

*CT* clotting time, *CFT* clot formation time, *Alpha angle* the angle of tangent between the centerline and the curve through the 20-mm amplitude point, *A5* amplitude at 5 min, *A10* amplitude at 10 min, *A20* amplitude at 20 min, *EXTEM* the extrinsic coagulation pathway, *INTEM* the intrinsic coagulation pathway, *FIBTEM* the quality of the fibrin-based clot, *APTEM* the detection of hyperfibrinolysis

After delivery, the baby (1173 g) showed respiratory distress. Tracheal intubation and administration of intratracheal lung surfactant were performed. One- and 5-min Apgar scores were 2 and 5, respectively. The neonatologists performed mechanical ventilation management for 2 days after delivery. The infant was discharged without further complications.

After delivery, continuous intravenous administration of propofol and remifentanil was started. Intraoperatively, 0.5 mg intravenous fentanyl was administered, and continuous fentanyl injection was started at 0.04 mg/h for postoperative analgesia. Intraoperative hemorrhage was 1813 mL (including amniotic fluid), and ascites was 1000 mL. The operation time was 61 min, and anesthesia time was 179 min. Urine volume was 80 mL, and infusion volume was 1954 mL (654 mL of crystalloids and 1300 mL of colloids). The patient also received 3 units of red blood cells and 6 units of fresh frozen plasma (FFP). The operation was completed without massive bleeding. However, a postoperative chest X-ray indicated pulmonary edema. We decided to continue mechanical ventilation in the intensive care unit (ICU). Albumin and diuretic administration improved the patient’s oxygenation and allowed for extubation the next day. She was transferred to the obstetrics ward 3 days postoperatively.

On the 15th postpartum day, her body weight showed a decrease of 20 kg, and she was discharged from the hospital. Subsequently, coagulation status was improved to pre-pregnancy levels 40 days after delivery.

## Discussion

WD shows abnormal copper accumulation in the liver and brain due to a decrease in ceruloplasmin, which is the transport protein bound to serum copper. ATP7B mutation on chromosome 13q14 gene has been identified as the causative gene [1, 2]. Generally, the treatment of WD is by pharmaceutical means that reduce copper absorption or promote copper excretion. Liver transplantation may also be performed in severe liver failure that is uncontrolled by pharmaceutical therapy.

Our patient had severe WD controlled with anti-copper therapy. Her liver function had rapidly deteriorated during pregnancy, and she showed severe coagulopathy, thrombocytopenia, and obstetric DIC. Intensive coagulation therapy could prevent massive peripartum hemorrhage.

Fibrinogen was reported to be the most important factor in obstetric hemorrhage [9]. It has been reported that a fibrinogen concentration ≤ 2 g/L has a positive predictive value of 100% for progression to severe postpartum hemorrhage [10]. Fibrinogen supplementation for postpartum hemorrhage has been reported [9–12]; however, prophylactic administration of fibrinogen in severe coagulopathy has not been well investigated. Our patient experienced both hypofibrinogenemia and thrombocytopenia, and was diagnosed with obstetric DIC. Her preoperative serum fibrinogen had decreased to < 1.5 g/L. Therefore, we considered the risk of massive perioperative hemorrhage to be high and that a massive blood transfusion may be required. The patient’s dramatic body weight gain had caused dyspnea and severe systemic edema. We were thus concerned about the cardiopulmonary dysfunction due to volume load caused by massive blood transfusion and fluid shift from prominent systemic edema. A large FFP transfusion can induce pulmonary edema due to volume overload [13]. Therefore, we decided to administer cryoprecipitate and fibrinogen concentrate. Additionally, we confirmed her coagulation function with ROTEM to determine the appropriate dose of fibrinogen concentrate. Although there have been reports about baseline ROTEM parameters of healthy pregnant women [14], ROTEM analysis in obstetrics has not been established. The efficacy of ROTEM-guided fibrinogen therapy for postpartum hemorrhage has been reported [15, 16]. In our case, her blood examination on the day of surgery showed thrombocytopenia, hypofibrinogenemia, and coagulation disorder. Preoperative platelet transfusion, cryoprecipitate, and fibrinogen concentrate administration improved her coagulation function and helped to avoid massive hemorrhage intraoperatively. Coagulation monitoring by ROTEM allowed us to evaluate her coagulation function just before surgery and determine the dose of supplemental fibrinogen administration.

WD exacerbation, as seen in our patient, is rare; therefore, we investigated other factors that can cause acute liver dysfunction associated with pregnancy, including the HELLP syndrome, acute fatty liver of pregnancy, and exacerbation of underlying chronic liver disease [17]. There are case reports of pregnant women with WD who had developed placenta abruption [18], preeclampsia [6], or HELLP syndrome [19, 20]. In our case, levels of total bilirubin, AST, and ALT were within normal limits. Therefore, liver function deterioration was considered to be due to WD exacerbation. However, the prominent systemic edema and significant weight gain might have also been attributed to a preeclampsia-related condition. Additionally, thrombocytopenia, decrease in urine output, and severe coagulopathy in the third trimester indicated obstetric DIC [12], which made her pregnancy difficult to continue.

Our patient had an episode of serious hepatic insufficiency at 20 years of age due to discontinuation of d-penicillamine. Discontinuation of medication in WD has been reported to exacerbate liver function and liver failure [21]. Liver biopsy revealed hepatic fibrosis, and liver function had already deteriorated severely enough to necessitate liver transplantation. Poor hepatic reserve may have been irreversible, despite copper metabolism and liver function showing improvement by treatment. She showed a remarkable decrease in liver function including albumin, AT-III, and coagulation factor levels, while AST and ALT levels remained normal. No reports show that only protein synthesis function markedly decreases in WD. Pregnancy might induce unexpected liver dysfunction in patients with WD, even when WD treatment is ongoing.

## Conclusion

We showed the successful management of pregnancy by cesarean delivery in a patient with WD. The exacerbation of WD during pregnancy caused severe coagulopathy, prominent body weight gain, respiratory distress, and placental insufficiency. Supplementation of coagulation factor before surgery allowed us to manage general anesthesia without massive bleeding. Both the mother and her baby were stable postoperatively, and the mother’s liver function recovered after delivery. Multidisciplinary therapy during pregnancy is critical for WD patients with liver failure. The appropriate continued treatment of WD, including pharmaceutical treatment and coagulation therapy, greatly increases the likelihood of a successful pregnancy, even in patients with liver failure exacerbation.

## Data Availability

Not applicable

## Abbreviations

ALT: Alanine aminotransferase
APTT: Activated partial thromboplastin time
AST: Aspartate aminotransferase
AT-III: Antithrombin III
CFT: Clot formation time
ChE: Cholinesterase
CS: Cesarean delivery
CT: Clotting time
DIC: Disseminated intravascular coagulation
FFP: Fresh frozen plasma
FGR: Fetal growth restriction
ICU: Intensive care unit
INR: International normalized ratio
POD: Postoperative day
PT: Prothrombin time
ROTEM: Rotational thromboelastometry
WD: Wilson’s disease
